# Clinical outcomes of immune checkpoint blockades and the underlying immune escape mechanisms in squamous and adenocarcinoma NSCLC

**DOI:** 10.1002/cam4.3590

**Published:** 2020-11-24

**Authors:** Yaru Tian, Xiaoyang Zhai, Weiwei Yan, Hui Zhu, Jinming Yu

**Affiliations:** ^1^ Department of Radiation Oncology Shandong Cancer Hospital and Institute Shandong University Jinan China; ^2^ Department of Radiation Oncology Shandong Cancer Hospital and Institute Shandong First Medical University and Shandong Academy of Medical Science Jinan China

**Keywords:** adenocarcinoma NSCLC, immune checkpoint blockades, immune escape mechanisms, squamous NSCLC

## Abstract

Immune checkpoint blockades (ICBs) have changed the standard of care of squamous and adenocarcinoma non‐small cell lung cancer (NSCLC). Whereas detailed researches regarding ICBs in the two major histological subtypes are rare. In order to uncover the clinical efficacy differences between squamous and adenocarcinoma NSCLC and better understand the underlying immune‐regulatory mechanisms, we compared the survival benefits of ICBs between the two subtypes by revealing phase 3 randomized trials and attempted to uncover the immune‐regulatory discrepancy. Generally, compared with nonsquamous NSCLC, squamous NSCLC benefited more from ICBs in Keynote 024, CheckMate 026, CheckMate 227 and CheckMate 017 and similar in OAK, but less in Keynote 010 and PACIFIC. We revealed that the tumor mutation burden (TMB) level, the programmed cell death ligand 1 (PD‐L1) expression, tumor infiltrating lymphocytes (TILs) in the tumor microenvironment (TME), chemokines, and oncogenic driver alterations within the two subtypes may contributed to the clinical outcomes of ICBs. We prospected that the combinations of ICBs with chemotherapy, radiation therapy, and antiangiogenic therapy could be promising strategies to re‐immunize the less immunogenic tumors and further enhance the efficacy of ICBs.

## Graphical abstract

We reviewed the clinical outcomes of immune checkpoint blockades and the underlying immune escape mechanisms in squamous and adenocarcinoma NSCLC, and prospected the strategies to improve the clinical efficacy of ICBs in NSCLC.

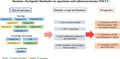

## 1 INTRODUCTION

Lung cancer remains the leading cause of cancer incidence and mortality in the world, with an estimated 2.1 million new cases and 18.4% of the total cancer‐related deaths in 2018.^1^ Non‐small cell lung cancer (NSCLC) accounts for approximately 85% of all lung cancers.^2^, ^3^ Over the past two decades, the increasingly understanding of the biology of NSCLC has revolutionized the treatment paradigm from traditional cytotoxic chemotherapy to personalized medicine, characterized by the development of small molecule tyrosine kinase inhibitors (TKIs) and immune checkpoint blockades (ICBs), based on the genetic alterations and the programmed cell death protein 1 (PD‐1) and its ligand (PD‐L1).^4^, ^5^ Anti‐PD‐1/PD‐L1 therapy was an approach to ‘‘immune normalization,” which selectively restored the tumor‐induced immune deficiency in the tumor microenvironment (TME) with fewer immune‐related adverse events (irAEs).^6^

The most common subtypes of NSCLC, squamous and adenocarcinoma NSCLC, have different origins. Basal cells in the proximal airway are considered to be the origin for squamous NSCLC, while adenocarcinoma NSCLC origins from type II pneumocytes, junction cells, and club cells of the bronchoalveolar duct.^7^, ^8^ As a result, the majority of genomic alterations are distinct between squamous (e.g., cyclin dependent kinase inhibitor 2A (*CDKN2A*) and tumor protein p53 (*TP53*)) and adenocarcinoma (e.g., KRAS proto‐oncogene (*KRAS*) and epidermal growth factor receptor (*EGFR*)) NSCLC.^7^ ICBs have dramatically altered the therapeutic landscape of advanced NSCLC. While the potential differences of immunotherapy between the two subtypes have not been fully evaluated yet. This review mainly discussed the clinical efficacy of ICBs and the dysfunctional immune microenvironment between squamous and adenocarcinoma NSCLC, and provided potential strategies to improve the clinical outcomes of immunotherapy.

## 2 CLINICAL OUTCOMES OF ICBS IN SQUAMOUS AND NONSQUAMOUS NSCLC.

The phase 3 randomized trials suggested that ICBs significantly improved the overall survival in patients with advanced squamous and nonsquamous NSCLC, but the clinical efficacy still varied between the two histological types.

### 2.1 First‐line

ICBs have revolutionized the first‐line treatment of advanced NSCLC. In Keynote 024,^9^, ^10^ pembrolizumab significantly prolonged progression‐free survival (PFS) and overall survival (OS) of patients with advanced NSCLC and PD‐L1 tumor proportion score (TPS) ≥50% (Table 1). And the OS improvement was more beneficial in squamous (hazard ratio (HR), 0.35; 95% confidence interval (CI), 0.17–0.71) than nonsquamous (0.55; 95% CI, 0.39–0.76) NSCLC. Keynote 042,^11^ which extended the patient population to PD‐L1 TPS ≥1%, also suggested that patients with TPS ≥50% benefited more from pembrolizumab than those with TPS 1–49% (HR for OS, 0.69 vs. 0.92, Table 1). Whereas nivolumab was not associated with significantly longer survival among patients with PD‐L1 TPS ≥5% in CheckMate 026^12^ (Table 1). But for the subgroup analysis, OS was more improved in squamous compared with nonsquamous NSCLC (HR, 0.82 vs. 1.17, Table 1).

**TABLE 1:** Large randomized phase 3 trials of ICBs in squamous and nonsquamous NSCLC

Study	N	Histology	Design	ORR	mPFS	mOS	HR for OS (95%CI)	PD‐L1 subgroup HR for OS (95%CI)
First‐line								
Keynote 024^9^, ^10^ PD‐L1≥50%	305	Squamous/ Nonsquamous	1. Pembro 2. Plat‐based Chemo	44.8% 27.8%	10.3 m 6.0 m	30.0 m 14.2 m	0.60 (0.41‐0.89)	—
Keynote 042^12^ PD‐L1≥1%	1274	Squamous/ Nonsquamous	1. Pembro 2. Plat‐based Chemo	27% 27%	5.4 m 6.5 m	16.7 m 12.1 m	0.81 (0.71‐0.93)	≥50%, 0.69 (0.56‐0.85) 1–49%, 0.92 (0.77‐1.11)
CheckMate 026^12^ PD‐L1≥5%	423	Squamous/ Nonsquamous	1. Nivo 2. Plat‐based Chemo	26% 33%	4.2 m 5.9 m	14.4 m 13.2 m	1.02 (0.80‐1.30)	≥50%, 0.90 (0.63‐1.29)
Keynote 407^13^	559	Squamous	1. Pembro+Pacl/Nab‐Pacl+Carbo 2. Placebo+Pacl/Nab‐Pacl+Carbo	57.9% 38.4%	6.4 m 4.8 m	15.9 m 11.3 m	0.64 (0.49‐0.85)	≥50%, 0.64 (0.37‐1.10) 1‐49%, 0.57 (0.36‐0.90) <1%, 0.61 (0.38‐0.98)
Keynote 189^14^	616	Nonsquamous	1. Pembro+Pem+Plat 2. Placebo+Pem+Plat	47.6% 18.9%	8.8 m 4.9 m	NR 11.3 m	0.49 (0.38‐0.64)	≥50%, 0.42 (0.26‐0.68) 1‐49%, 0.55 (0.34‐0.90) <1%, 0.59 (0.38‐0.92)
IMpower131^15^	683	Squamous	1. Atezo+Nab‐Pacl+Carbo 2. Nab‐Pacl+Carbo	49% 41%	6.3 m 5.6 m	14.0 m 13.9 m	0.96 (0.78‐1.18)	HR for PFS TC3 or IC3, 0.44 (0.27‐0.71) TC1/2 or IC1/2, 0.70 (0.53‐0.92) TC0 and IC0, 0.81 (0.64‐1.03)
IMpower132^16^	578	Nonsquamous	1. Atezo+Pem+Plat 2. Placebo+Pem+Plat	47% 32%	7.6 m 5.2 m	18.1 m 13.6 m	0.81 (0.64‐1.03)	HR for PFS TC3 or IC3, 0.46 (0.22‐0.96) TC1/2 or IC1/2, 0.80 (0.56‐1.16) TC0 and IC0, 0.45 (0.31‐0.64)
IMpower150^17^	800	Nonsquamous	1. Atezo+Bev+Pacl+Carbo 2. Bev+Pacl+Carbo	63.5% 48.0%	8.3 m 6.8 m	19.2 m 14.7 m	0.78 (0.64‐0.96)	TC3 or IC3, 0.39 (0.25‐0.60) TC1/2 or IC1/2, 0.56 (0.41–0.77) TC0 and IC0, 0.77 (0.61–0.99)
CheckMate 227^18^	1166	Squamous/ Nonsquamous	1. Nivo+ipi 2. Chemo	‐	‐	17.1 m 13.9 m	0.73 (0.64–0.84)	≥50%, 0.70 (0.55–0.90) 1–49%, 0.94 (0.75–1.18) <1%, 0.62 (0.49–0.79)
Second‐line and beyond								
Keynote 010^19^ PD‐L1≥1%	1034	Squamous/ Nonsquamous	1. Pembro 2 mg/kg 2. Pembro 10 mg/kg 3. Docetaxel	18% 18% 9%	3.9 m 4.0 m 4.0 m	10.4 m 12.7 m 8.5 m	1:3 0.71 (0.58–0.88) 2:3 0.61 (0.49–0.75)	≥50%, 0.53 (0.40–0.70) 1–49%, 0.76 (0.60–0.96)
OAK^20^	1225	Squamous/ Nonsquamous	1. Atezo 2. docetaxel	14% 13%	2.8 m 4.0 m	13.8 m 9.6 m	0.73 (0.62–0.87)	TC3 or IC3, 0.41 (0.27–0.64) TC1/2/3 or IC1/2/3, 0.74 (0.58–0.93) TC0 and IC0, 0.75 (0.59–0.96)
CheckMate 017^21^	272	Squamous	1. Nivo 2. Docetaxel	20% 9%	3.5 m 2.8 m	9.2 m 6.0 m	0.59 (0.44–0.79)	≥10%, 0.50 (0.28–0.89) ≥5%, 0.53 (0.31–0.89) ≥1%, 0.69 (0.45–1.05)
CheckMate 057 ^22^	582	Nonsquamous	1. Nivo 2. Docetaxel	19% 12%	2.3 m 4.2 m	12.2 m 9.4 m	0.73 (96%CI, 0.59–0.89)	‐
**Consolidation**								
PACIFIC^23^, ^24^	709	Squamous/ Nonsquamous	1. CRT+Durva 2. CRT+Placebo	28.5% 20%	17.2 m 5.6 m	NR 28.7 m	0.68 (99.73%CI, 0.47–0.997)	HR for PFS ≥25%, 0.41 (0.26–0.65) <25%, 0.59 (0.43–0.82)

Abbreviations: Atezo, atezolizumab; Bev, bevacizumab; Carbo, carboplatin; Chemo, chemotherapy; CI, confidential interval; CRT, chemoradiotherapy; Durva, durvalumab; HR, hazard ratio; Nab‐Pacl, nanoparticle albumin‐bound–paclitaxel; Nivo, nivolumab; ORR, objective response rate; OS, overall survival; Pacl, paclitaxel; Pem, pemetrexed; Pembro, pembrolizumab; PFS, progression‐free survival; Plat, platinum.

The combination of ICBs and chemotherapy has made a synergistic effect in treating advanced NSCLC. Compared with chemotherapy, the addition of pembrolizumab resulted in greatly improved OS for squamous and nonsquamous NSCLC in Keynote 407^13^ and Keynote 189^14^ (Table 1). OS improvements were similar among PD‐L1 subgroups in Keynote 407, but increased with PD‐L1 expression in Keynote 189 (HR, 0.59, 0.55 and 0.42 for TPS <1%, 1%–49% and ≥50%, respectively). Moreover, atezolizumab plus chemotherapy significantly prolonged PFS in squamous and nonsquamous NSCLC (Table 1). For squamous NSCLC in IMpower131,^15^ PFS in the high PD‐L1 (PD‐L1 expression of tumor cell (TC) or immune cell (IC), (TC3 or IC3)) group benefited more from the combination therapy than the low (TC1/2 or IC1/2) or negative (TC0 and IC0) groups (Table 1). Whereas for nonsquamous NSCLC in IMpower132,^16^ PFS was more prolonged in the high and negative groups compared with the low group (Table 1). The risk of disease progression or death also decreased with the addition of atezolizumab to bevacizumab and chemotherapy across high, low and negative PD‐L1 groups in IMpower150^17^ (Table 1). Teff gene‐signature could also predict clinical benefit of atezolizumab. CheckMate 227^18^ suggested that the first‐line treatment of double ICBs (nivolumab plus ipilimumab) resulted in prolonged OS, independent of PD‐L1 expression (Table 1). Among PD‐L1 positive subgroup, squamous NSCLC had a lower risk of death than nonsquamous NSCLC.

### 2.2 Second‐line

For the second‐line setting, Keynote 010^19^ suggested that pembrolizumab significantly improved the objective response rate (ORR), PFS and OS among patients with advanced NSCLC and PD‐L1 TPS ≥1% (Table 1). And OS favored pembrolizumab more in TPS ≥50% group than 1%–49% group (HR 0.53 vs. 0.76, Table 1). However, squamous NSCLC benefited less from immunotherapy than nonsquamous NSCLC (HR, 0.74 vs. 0.63, Table 1). Atezolizumab resulted in a relevant improvement of OS versus docetaxel regardless of PD‐L1 expression or histology in OAK^20^ (Table 1). The OS improvements were similar between squamous and nonsquamous NSCLC (HR, 0.73 vs. 0.73, Table1). Nivolumab was also associated with improved ORR, PFS and OS among patients with advanced squamous and nonsquamous NSCLC in CheckMate 017^21^ and CheckMate 057^22^ (Table 1). PD‐L1 expression at the cutoff value of 5% and 10% could predict the efficacy of nivolumab. In CheckMate 017, the risk of death was 41% lower with nivolumab than with docetaxel in squamous NSCLC. Whereas it was only 27% lower in patients with nonsquamous NSCLC in CheckMate 057.

### 2.3 Consolidation

PACIFIC^23^, ^24^ was a phase 3 study comparing durvalumab as consolidation therapy with placebo in patients with stage III NSCLC who did not have disease progression after ≥2 cycles of the first‐line regimens and radiotherapy. PFS and OS favored durvalumab compared to chemotherapy (Table 1). The decrease of the risk of disease progression or death decreased was more among patients with PD‐L1 expression ≥25% than those with PD‐L1 <25% (HR, 0.41 vs. 0.59). The clinical benefit was more favorable in nonsquamous than squamous NSCLC.

These clinical trials clearly demonstrate that immunotherapy has brought about remarkable survival benefit in advanced NSCLC. PD‐L1 expression is a reliable biomarker to predict the clinical efficacy of ICBs. The survival benefit was more favorable in squamous than nonsquamous type in some studies (Keynote 024, CheckMate 026, CheckMate 227, CheckMate 017), and was similar in OAK, but was less favorable in Keynote 010 and PACIFIC. Considering the potential clinical efficacy differences between the two histologic types, we further explored the underlying immunologic mechanisms.

## 3 IMMUNE ESCAPE MECHANISMS IN SQUAMOUS AND ADENOCARCINOMA NSCLC

### 3.1 Tumor mutation burden

The overall tumor mutation burden (TMB) of NSCLC was 8.0 mutations/megabase (Mb) and TMB was significantly lower in adenocarcinoma compared with squamous NSCLC (*p* = 0.024).^25^ In Chinese patients with early stage squamous NSCLC, Jiang T et al.^26^ identified that the median TMB was 9.43 mutations/Mb. Consistently, neoantigens was higher in squamous than adenocarcinoma NSCLC, with 53% of the squamous tumors and 47% of adenocarcinoma tumors harboring at least five predicted neoepitopes.^27^ In addition, patients with *EGFR* mutations, especially the sensitive subtype, had a significantly decreased TMB level than those with *EGFR* wide type (*WT*) (median 3.77 vs. 6.12 mutations/Mb), displaying a low immunogenicity.^28^, ^29^ As a less invasive method, blood‐based TMB (bTMB) was reported to have a good correlation with tissue TMB (tTMB) and could also predict tumor responses to ICBs in patients with NSCLC.^30^, ^31^, ^32^ Most data to date is retrospective and there are several panels to determine bTMB. Furthermore, studies on bTMB between squamous and nonsquamous NSCLC are rare, which need further investigations.

### 3.2 PD‐L1 expression

PD‐L1 expression is an effective biomarker to predict the clinical efficacy of ICBs according to phase 3 randomized trials. To better understand the underlying mechanisms, we then explored PD‐L1 expression in squamous and nonsquamous or adenocarcinoma NSCLC. PD‐L1 expression may vary from detecting antibodies, IHC methods or expression cells (TCs or ICs). Table 2 compared PD‐L1 expression from multiple clinical studies between the two histologic types. Keynote 407^13^ and Keynote 189^14^ indicated that PD‐L1 expression was similar between the two subtypes. Under the Dako PD‐L1 22C3 pharmDx platform, the prevalence of PD‐L1 expression was higher in squamous NSCLC. Yu H et al.^33^ found that 46.7% and 61.5% of squamous specimens had positive PD‐L1 expression on TCs and ICs. While Shinchi Y et al.^34^ only detected a positive PD‐L1 expression rate of 26.8% in adenocarcinoma NSCLC. Two retrospective analyses also indicated a higher percentage of PD‐L1 expression in squamous than adenocarcinoma NSCLC (72.3% vs. 36.9% and 34.3% vs. 4.1%, respectively).^35^, ^36^ The SP142 PD‐L1 IHC assay (Ventana) detected that the prevalence of PD‐L1 expression ranged from 50% to 55% in squamous NSCLC,^15^, ^25^, ^37^, ^38^, ^39^ but was almost less than 40% in adenocarcinoma NSCLC.^16^, ^25^, ^39^ And the positive PD‐L1 expression rate was 83% in squamous NSCLC (CheckMate 017)^21^ versus 78% in nonsquamous NSCLC in (CheckMate 057).^22^ Mazzaschi G et al.^30^ found that squamous NSCLC specimens exhibited a 2.5‐fold higher PD‐L1 value than adenocarcinoma cases. Kim S et al.^40^ detected that PD‐L1 positivity was observed in 28.1% of adenocarcinomas. The prevalence of PD‐L1 TPS ≥5% was detected to be higher in squamous than adenocarcinoma NSCLC in two retrospective studies (31% vs. 23% and 28% vs. 20%, respectively).^41^, ^42^ And the overall frequency of PD‐L1 expression was 56.2% and 39.9% on TCs of squamous and adenocarcinoma NSCLC, respectively.^43^, ^44^ What's more, multivariate analysis indicated that high PD‐L1 expression was independently associated with squamous histology and smokers.^45^, ^46^, ^47^, ^48^

**TABLE 2:** PD‐L1 expression in squamous and nonsquamous/adenocarcinoma NSCLC

Study	PD‐L1 antibody	PD‐L1 expression	N	Squamous	N	Nonsquamous/Adeno
Keynote 407^13^	22C3/Agilent	TC	559	63.1%		
Keynote 189^14^	22C3/Agilent	TC			616	63.0%
Yu H et al^33^	22C3/Dako	TC	255	46.7%		
		IC		61.5%		
Shinchi Y et al^34^	22C3/Dako	TC			231	26.8%
Lee SE et al^35^	22C3/Dako	TC	188	72.3%	785	36.9%
Pan Y et al^36^	22C3/Dako	TC	108	34.3%	221	4.1%
IMpower131^15^	SP142/Ventana	TC/IC	683	51.4%		
Takada K et al^37^	SP142/Ventana	TC	202	52.5%		
Takada K et al^38^	SP142/Ventana	TC/IC	205	51.7%		
IMpower132^16^	SP142/Ventana	TC/IC			578	31.3%
IMpower150^17^	SP142/Ventana	TC/IC			800	51%
Chen Y et al^25^	SP142/Ventana	TC/IC	51	55%	136	37%
Janzic U et al^39^	SP142/Ventana	TC/IC	25	52% (≥5%)	29	17% (≥5%)
CheckMate 017^21^	clone 28–8/Dako	TC	272	83%		
CheckMate 057^22^	clone 28–8/Dako	TC			582	78%
Kim S et al^40^	E1L3N/CST	TC			146	28.1%
Parra ER et al^42^	E1L3N/CST	TC	108	31% (≥5%)	146	23% (≥5%)
Schmidt LH et al^41^	E1L3N/CST	TC	149	28% (≥5%)	125	20% (≥5%)
Yang CY et al^43^, ^44^	Polyclonal Ab/ Proteintech	TC	105	56.2%	163	39.9%

These studies consistently suggested that the prevalence of PD‐L1 expression was significantly higher in squamous than adenocarcinoma NSCLC, which may explain the better result of ICBs in squamous NSCLC in part.

### 3.3 Tumor infiltrating lymphocytes and chemokines

Tumor infiltrating lymphocytes (TILs) and chemokines play an important role in regulating immune response. Thus, we attempted to find out the immune microenvironment in the two NSCLC subtypes.

The immune cells were less functional in adenocarcinoma than squamous NSCLC. Kinoshita T et al.^49^ determined that the insufficiently activated infiltrating CD8+ T cells, immune‐regulatory CD8+FOXP3+T cells and immune‐dysfunctional CD8+GATA3+ T cells contributed to the immunosuppressive microenvironment in non‐smokers with adenocarcinoma. Additionally, the enrichment of Foxp3+ Tregs was associated with a drastic decrease of NK cells in adenocarcinoma samples and metastatic lymph nodes. In contrast, squamous carcinomas displayed less profound accumulation of Tregs.^50^ In elderly patients with adenocarcinomas, despite of the increased number of CD8+ T cells, the expressions of cytolytic molecule (granzyme B, perforin 1, granzyme A, granzyme M, and granulysin) were impaired, which was associated with a loss of clonal neoantigens. A number of immunosuppressive elements were upregulated, including Treg cells and co‐inhibitory molecules (e.g., T cell immunoglobulin and mucin domain‐containing protein‐3 (*TIM*‐*3*), T cell immunoreceptor with Ig and ITIM domains (*TIGIT*), and HERV‐H LTR‐associating 2 (*HHLA2*)).^51^ *HHLA2*, a newly discovered member of B7 family, was associated with EGFR mutation and was higher in lung adenocarcinoma compared with squamous NSCLC.^52^ A high FOXP3/CD4 ratio and a low number of CD20+ B cells were identified as negative prognostic factors in adenocarcinomas.^53^ The lack of memory B cells or increased M0 macrophages in adenocarcinoma NSCLC were correlated with the poor prognosis. Whereas T follicular helper cells in squamous NSCLC were associated with favorable prognosis.^54^ A significant 2.5‐fold higher average PD‐L1 expression, three‐fold increase in CD57+ cytotoxic cells and 1.5‐fold increase in PD‐1+ lymphocytes was detected in squamous samples compared to adenocarcinomas.^55^ A high level of intraepithelial CD45RO+ TILs in lymph‐node metastases was an independent positive prognostic factor for PFS in squamous NSCLC, but not in adenocarcinoma NSCLC patients.^56^

Chemokines play an important part in regulating immune function in TME. In adenocarcinomas, bone morphogenetic protein‐4 (BMP4), one of the tumor‐derived regulatory programs, could augment PD‐L1 expression in the mesenchymal subset of lung cancer cells.^57^ Adenocarcinomas had higher levels of MCP1/CCL2 and MIP‐1β/CCL4 than squamous NSCLC. CCL2 and CCL4 overexpression was associated with beneficial OS and PFS in squamous NSCLC, but unfavorable OS and PFS in adenocarcinoma NSCLC.^52^ What's more, glycogen branching enzyme (GBE1) was also involved in the immune dysregulation in adenocarcinoma NSCLC. GBE1 blockade promoted the secretion of CCL5 and CXCL10 to recruit CD8+ T cells to the TME via the *IFN*‐*I*/*STING* signaling pathway.^58^

### 3.4 ICBs in patients with oncogenic driver mutations

Adenocarcinoma NSCLC is characterized of high prevalence of oncogenic driver mutations, with *EGFR* mutation rate of 27% and anaplastic lymphoma kinase (*ALK*) rearrangement rate of <8%.^4^ However, most clinical trials excluded patients with sensitizing *EGFR* or *ALK* mutations. Table 3 summarized studies evaluating clinical outcomes of ICBs in *EGFR*‐mutant population.

**TABLE 3:** Clinical outcomes of ICBs in EGFR‐mutant population

Study	Design	N	Clinical outcome
Keynote 010^19^	1. Pembro 2. Docetaxel	EGFR WT, 875 EGFR Mutation, 86	HR for OS, 95%CI 0.66; 0.55–0.80 vs. 0.88; 0.45–1.70
OAK^20^	1. Atezo 2. docetaxel	EGFR WT, 628 EGFR Mutation, 85	HR for OS, 95%CI 0.83;0.58–1.18 vs. 1.24; 0.71–2.18
CheckMate 057^22^	1. Nivo 2. Docetaxel	EGFR WT, 340 EGFR Mutation, 82	HR for OS, 95%CI 0.66; 0.51–0.86 vs. 1.18; 0.69–2.00
PACIFIC^23^	1. CRT+Durva 2. CRT+Placebo	EGFR WT, 481 EGFR Mutation, 43	HR for PFS, 95%CI 0.47; 0.36–0.60 vs. 0.76; 0.35–1.64
Lisberg A et al^59^ Phase 2 trial	Pembro	EGFR Mutation, 11	1 case had ORR
Dong ZY et al^28^ A pool‐analysis	1. ICBs (pembro, nivo or atezo) 2. Docetaxel	EGFR WT, 1990 EGFR Mutation, 271	HR for OS, 95%CI 0.67; 0.61–0.76 vs. 1.09; 0.84–1.41
Lee CK et al^61^. A meta‐analysis	1. ICBs (pembro, nivo or atezo) 2. Docetaxel	EGFR WT, 1362 EGFR Mutation, 186	HR for OS, 95%CI 0.66; 0.58–0.76 vs. 1.05; 0.70–1.55
Cho JH et al^62^ Retrospective	ICBs (pembro or nivo)	EGFR WT, 140 EGFR Mutation, 38	ORR, 32.9% vs. 15.8%
Hastings K et al^63^ Retrospective	ICBs	EGFR WT, 212 EGFR 19 deletion, 80 EGFR L858R, 46	ORR, 22% vs. 7% vs. 16% HR for OS, 95%CI 19 deletion vs. WT 0.69, 0.493–0.965 L858R vs. WT 0.917, 0.597–1.409,
Yamada T et al^64^ Retrospective	ICBs	EGFR Mutation, 27 Uncommon, 7 Common, 20	ORR, 57% vs. 7% DCR, 71% vs. 35.7%

Abbreviations: Atezo, atezolizumab; CI, confidential interval; CRT, chemoradiotherapy; DCR, disease control rate; Durva, durvalumab; HR, hazard ratio; Nivo, nivolumab; ORR, objective response rate; OS, overall survival; Pembro, pembrolizumab; WT, wide type.

Keynote 010^19^ indicated that the *EGFR WT* population significantly benefited from ICBs compared with the *EGFR*‐mutant population (HR for OS, 0.66 vs. 0.88). The subgroup analysis of OAK^20^ suggested an improved survival in the *EGFR WT* population (HR, 0.83; 0.58–1.18), but a worse survival in the *EGFR*‐mutant population (HR, 1.24; 0.71–2.18). The positive *EGFR* mutation was also a negative prognostic factor in CheckMate 057^22^ (HR, 1.18; 0.69–2.00). What's more, consolidation durvalumab remarkably decreased the risk of disease progression in locally advanced NSCLC patients without sensitizing *EGFR* mutations (HR, 0.47; 0.36–0.60) but not in those with mutations (HR, 0.76; 0.35–1.64).^23^ A phase 2 study revealed that the first‐line pembrolizumab lacked efficacy in PD‐L1+, *EGFR*‐mutant patients.^59^ In the real‐world practice, *EGFR* mutation or *ALK* rearrangement was an independent negative predictor of OS in patients treated with anti‐PD‐1 therapy.^60^ A pool‐analysis of four randomized control trials confirmed that patients with *EGFR WT*, but not *EGFR* mutation, could benefit from PD‐1/L1 inhibitors.^28^ A meta‐analysis demonstrated that ICBs significantly prolonged OS in the *EGFR* WT subgroup (HR, 0.66; 0.58–0.76) but not the *EGFR*‐mutant subgroup.^61^ What's more, Cho JH et al.^62^ found that the *EGFR*‐mutant group receiving ICBs had a lower ORR than the *EGFR WT* group (15.8% vs. 32.9%). In addition, Hastings K et al.^63^ explored the heterogeneity of *EGFR*‐mutant tumors and found that compared with 212 *EGFR WT* tumors, the clinical outcomes with PD‐L1 blockade were worse in patients harboring *EGFR* exon 19 deletion, but similar in those with *EGFR* L858R mutation. They also demonstrated that this difference was due to a lower TMB in tumors with *EGFR* exon 19 deletion than those with *EGFR* L858R mutation. Yamada T et al.^64^ enrolled 27 patients with *EGFR*‐activating mutations and confirmed a higher ORR and DCR in patients with uncommon *EGFR* mutations than those with common *EGFR* mutations (71% vs. 35.7% and 57% vs. 7%). Moreover, *EGFR* mutation patients without T790 M mutation were more likely to benefit from nivolumab, possibly because of a higher PD‐L1 expression than those with T790 M mutation.^65^

In contrast to *EGFR* mutations and *ALK* rearrangement, patients with *KRAS* mutation seemed to achieve more benefit from ICBs. OS was significantly improved in *KRAS*‐mutant subgroup receiving nivolumab in CheckMate 057^22^ (HR, 0.52; 0.29–0.95). In OAK,^20^ patients with *KRAS* mutation benefited more from atezolizumab (median OS, 17.2 m vs. 10.5 m; HR, 0.71; 0.38–1.35) than those with *KRAS WT* (13.8 m vs. 11.3 m; HR, 0.83; 0.58–1.18). Clinical activity of ICBs was higher in the *KRAS* group (ORR 26%; median PFS, 3.2 m) than the *EGFR* (12%; 2.1 m), *BRAF* (24%; 3.1 m) and *MET* (16%; 3.4 m) group, and even lacked response in the *ALK* group.^66^ Another study elucidated that the favorable outcome of ICBs in *BRAF* mutants was probably due to a high PD‐L1 expression.^67^ Even though a proportion of tumors with *MET* exon 14 mutation had PD‐L1 expression, the median TMB was lower than unselected patients, and clinical efficacy is modest.^68^

### 3.5 Immune escape mechanisms in *EGFR*‐mutant population

Considering the lack of clinical efficacy of ICBs in patients with positive *EGFR* mutations, the underlying immune escape mechanisms need to be clarified.

Multiple studies have confirmed that PD‐L1 expression was associated with *EGFR* status.^35^, ^48^, ^69^, ^70^, ^71^, ^72^, ^73^ Patients with *EGFR* mutations had decreased PD‐L1 expression according to a pool‐analysis of 15 public studies.^28^ And this inverse correlation between *EGFR* mutation and PD‐L1 expression was also confirmed from the analyses of The Cancer Genome Atlas (TCGA) and Guangdong Lung Cancer Institute (GLCI) cohort.^28^ Rangachari D et al.^74^ found that PD‐L1 TPS ≥50% seldom overlapped with driver oncogenes. A retrospective study in Japan only detected a 9.9% (seven of 71) TPS ≥50% rate among *EGFR*‐mutant patients.^75^ Another Japanese study revealed that 23.9% (11 of 46) patients with TPS ≥50% had positive *EGFR* mutations.^76^ Gainor JF et al.^77^ also indicated that ORR was significantly lower in *EGFR*‐mutant or *ALK*‐positive patients (3.6%) than *EGFR* and *ALK*‐*WT* patients (23.3%). The underlying mechanisms may involve in the low rate of concurrent PD‐L1 expression and CD8+ TILs within the TME. Liu SY et al.^78^ detected a lower proportion of PD‐L1+/CD8+ tumors in patients with *EGFR* mutation or *ALK* rearrangement (5.0%, 17/342) than those with *EGFR* and *ALK WT* (14.2%, 45/316). Dong ZY et al.^28^ also discovered a lack of T‐cell infiltration and shrinking proportion of CD8+ TILs in *EGFR*‐mutant population. In addition, *EGFR* activation probably contributed to the uninflamed TME and participated in immunosuppression and immune escape via generation of Tregs, tolerogenic dendritic cells (DCs), and myeloid‐derived suppressor cells (MDSCs).^79^ The EGF‐like growth factor Amphiregulin enhanced Tregs suppressive function via the *EGFR*/*GSK*‐*3*/*Foxp3* axis.^80^, ^81^ Activating signal transducer and activator of transcription 3 (*STAT3*), a downstream signaling molecule of *EGFR*, inhibited DCs maturation,^82^ and promoted MDSC‐mediated immunosuppression.^83^

The immune microenvironment was consistent with the distinct immune response of *EGFR*‐ and *KRAS*‐mutant patients.^84^ Huynh TG et al demonstrated that concurrent PD‐L1 expression and abundant CD8+ TILs were observed in 25% of *KRAS* mutants or cases without alterations versus only 7.4% of *EGFR* mutants.^73^ In contrast to the low immune infiltration associated with *EGFR* mutations, *KRAS* mutations were significantly associated with T cell infiltration.^85^ Although lymphocytes were present in TME, *EGFR*‐mutant tumors had a high frequency of inactive TILs. While TILs in *KRAS* mutants were almost active, inflamed with higher CD4+, CD8+, and CD20+ TILs. They also revealed that activated *EGFR* correlated with increased PD‐L1 expression in *EGFR* mutants but not in *EGFR WT*, whereas TIL activation was associated with higher PD‐L1 only in *EGFR*/*KRAS WT*. Thus, PD‐L1 may reflect the constitutive oncogenic signaling in *EGFR* mutants rather than immune signaling in *EGFR WT*, which would be associated with high PD‐L1 levels and TILs activation.

## 4 PROSPECTIVE

Adenocarcinoma NSCLC seemed to be more immunosuppressive and benefited less from ICBs than squamous NSCLC. Strategies to improve the clinical efficacy of ICBs in adenocarcinoma is in need.

### 4.1 Combination of ICBs and immunogenic drugs and radiation therapy

Compared with monotherapy, the combination of ICBs and chemotherapy has improved the clinical efficacy in patients with nonsquamous NSCLC in Keynote 189^14^ and IMpower132.^16^ Using immunogenic drugs (oxaliplatin, cyclophosphamide et al), lung adenocarcinoma tumors that lacked T‐cell infiltration and resisted current treatments in mouse models could be successfully sensitized to host antitumor T‐cell immunity and further response to ICBs.^86^ Combing ICBs with chemotherapy could enhance the recognition and elimination of tumor cells by the host immune system and refine the immunosuppressive TME.^87^ Radiation therapy also had a synergistic effect with immunotherapy via enhancing MHC class I expression, activating DCs and promoting cross‐presentation of tumor antigens, increasing the density of TILs, modulating the expression of immune checkpoint molecules, modulating Treg populations et al.^88^, ^89^ Consolidation durvalumab after concurrent chemoradiotherapy has brought about significantly prolonged OS in phase III advanced NSCLC.^22^ In addition, the PEMBRO‐RT phase 2 randomized clinical trial demonstrated that the high‐dose stereotactic body radiotherapy (SBRT) on a single tumor site prior to pembrolizumab remarkably enhanced tumor response in patients with metastatic NSCLC. Thus, for those tumors uninflamed with active TILs and less immunogenic, chemotherapy or radiation therapy could be effective strategies to enhance the anti‐tumor activities of ICBs.

### 4.2 Combination of ICBs and antiangiogenic therapy

Angiogenesis was considered as one of the hallmarks of cancer.^90^ Vascular endothelial growth factor (*VEGF*), the major regulator in tumor angiogenesis, contributed to immune escape via blocking DC differentiation, inhibiting T‐cell development and reducing its infiltration, inducing Tregs and MDSCs et al.^91^, ^92^ In IMpower150,^17^ the addition of atezolizumab to bevacizumab and chemotherapy significantly prolonged OS in patients with advanced nonsquamous NSCLC. Based on the ALTER 0303 study,^93^ the Chinese Food and Drug Administration (CFDA) approved anlotinib, an anti‐angiogenesis tyrosine multikinase inhibitor, as a third‐line or later therapy for advanced NSCLC. Therefore, antiangiogenic therapy can be synergistic with immunotherapy and improve the clinical efficacy of ICBs.

### 4.3 Combination of ICBs and *EGFR*‐TKIs

Multiple clinical trials are exploring the effect of the combinations of *EGFR*‐TKIs and *ALK*‐TKIs with ICBs, the results are immature.^94^ The phase 1/2 KEYNOTE‐021 study suggested that pembrolizumab plus erlotinib did not improve ORR compared with previous monotherapy studies for patients with advanced NSCLC and sensitizing EGFR mutation.^95^ And the high incidence of treatment‐related toxicities associated with these combinations made this approach more controversial.^96^

## 5 CONCLUSION

Large randomized trials have confirmed the extraordinary effects of ICBs in advanced NSCLC. Squamous NSCLC may benefit more from ICBs than adenocarcinoma NSCLC in considerations of the high TMB, high PD‐L1 expression, more functional TILs in the TME and chemokines. In addition, tumors with active driver mutations, especially EGFR mutations, had more uninflamed and immunosuppressive TME and responded less for ICBs. We prospected that chemotherapy, radiation therapy, and antiangiogenic therapy may be promising to enhance the antitumor activity of ICBs.

## 6 COMPETING INTERESTS

The authors declare that they have no competing interests.

## AUTHORS’ CONTRIBUTION

Jinming Yu and Hui Zhu conceived and designed the study; Yaru Tian wrote the paper; Xiaoyang Zhai and Weiwei Yan coordinated on the study.

## ETHICS APPROVAL AND CONSENT TO PARTICIPATE

This article does not contain any studies with human participants or animals.

## Data Availability

No additional data are contained in this review.
